# *PhyloRef*: A Semi‐Automated Workflow for eDNA Reference Database Curation via Phylogenetic Anomaly Detection

**DOI:** 10.1002/ece3.73159

**Published:** 2026-02-26

**Authors:** Yan Mai, Chenhong Li

**Affiliations:** ^1^ Shanghai Universities Key Laboratory of Marine Animal Taxonomy and Evolution Shanghai Ocean University Shanghai China; ^2^ Engineering Research Center of Environmental DNA and Ecological Water Health Assessment Shanghai Ocean University Shanghai China

**Keywords:** Actinopterygii, Chondrichthyes, environmental DNA, phylogenetic anomaly, reference database, species misidentification

## Abstract

Environmental DNA (eDNA) analysis depends critically on high‐quality reference databases. However, widely used public repositories (e.g., NCBI) frequently suffer from annotation error, species misidentification, and sequence contamination, leading to unreliable biodiversity assessments. To address these issues, we introduce *PhyloRef*, a Snakemake‐based, semi‐automated phylogeny‐guided workflow for reference library curation. *PhyloRef* improves scalability via taxonomic grouping, detects problematic records using clustering‐based anomaly detection rather than rigid monophyly requirements, and conservatively flags ambiguous cases using a “*similar_to=*” annotation. *PhyloRef* leverages complete mitochondrial genomes while flexibly incorporating single‐gene sequences to maximize taxonomic coverage when complete genomes are scarce. The workflow categorizes anomalies into three types: (1) single‐sequence outliers, (2) inconsistent sequence pairs, and (3) minority deviations within multi‐sequence clusters, flagging them for manual review via convenient visualizations or deleting them automatically by option. Importantly, sequences with ambiguous phylogenetic placement are annotated with a “*similar_to=*” label to alert users to potential uncertainty. We validated *PhyloRef* using mitochondrial genome datasets for Chondrichthyes (cartilaginous fishes) and Actinopterygii (ray‐finned fishes) extracted from NCBI. The tool identified and removed nine anomalous chondrichthyan sequences and 401 Actinopterygian sequences (~2.3% and ~5.2% of the initial datasets, respectively), yielding curated databases of 380 sequences (266 species) and 7258 sequences (4887 species), respectively. In addition, nine sequences were flagged with “*similar_to=*” label in chondrichthyan fishes and 597 in Actinopterygian fishes, to reduce the risk of misidentification in downstream eDNA analyses. This resource enhances the reliability of eDNA‐based biodiversity and ecological studies. Future directions include integrating machine learning for anomaly detection, incorporating nuclear markers for improved taxonomic resolution, and developing automated updating modules.

## Graphical abstract

*PhyloRef* processes complete mitochondrial genomes with optional multi‐gene concatenation to flag three categories of phylogenetic anomalies based on tree topology and annotates ambiguous sequences with “similar_to=” labels. The workflow successfully curated 7600+ chondrichthyan and actinopterygian sequences from NCBI, identifying and removing 410 anomalous entries to enhance database reliability.

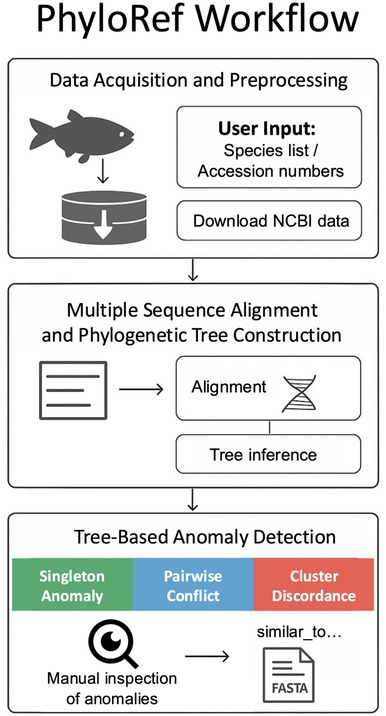

## 1 Introduction

Environmental DNA (eDNA)‐based approach has recently attracted considerable attention due to its broad applicability in biodiversity monitoring, invasive species detection, and conservation of endangered species (Duhamet et al. 2023; Hoban et al. 2022; Jackman et al. 2021). By detecting and analyzing trace DNA from environmental samples, such as air, soil, or water, eDNA enables non‐invasive assessments of species composition and distribution in ecosystems. Compared with traditional biological monitoring approaches, eDNA‐based analyses offer several advantages: easy sampling, reduced cost, minimal ecological disturbance, and no requirement for specialized taxonomic expertise (Xiong et al. 2022).

Despite these benefits, practical applications of eDNA still face several challenges, especially regarding the quality control of reference databases (Keck and Altermatt 2023). Widely used public databases such as GenBank and BOLD often contain misannotated sequences or entries with insufficient taxonomic resolution (Keck, Brantschen, and Altermatt 2023; Keck, Couton, and Altermatt 2023; Steinegger and Salzberg 2020), including species misclassification, merging sequences from distinct species, inconsistent nomenclature (e.g., use of vernacular instead of standardized Latin names), and limited discriminatory power of short sequence fragments (Dziedzic et al. 2023; Ratnasingham and Hebert 2007; Steinegger and Salzberg 2020). These problems may arise from misidentification during sampling, data entry errors during submission, PCR contamination, or the intrinsic limitations of certain molecular markers (Dziedzic et al. 2023). Despite increasing awareness, a practical gap remains: many QC approaches are either rule‐based filters that can miss phylogenetically inconsistent records or phylogeny‐based checks that are difficult to scale and challenging to reproduce across large datasets. Consequently, there is a need for a scalable and transparent workflow that integrates phylogenetic validation with standardized reporting of anomalies, while retaining informative but ambiguous records when appropriate.

A striking example involves the European anchovy (*Engraulis encrassicolus*), whose sequences have been erroneously assigned to the Atlantic sailfish ( *Istiophorus albicans* ) in public databases (Claver et al. 2023). These two species exhibit starkly different distribution patterns in the Bay of Biscay; the European anchovy maintains a large population and serves as a keystone species in the local ecosystem, while the Atlantic sailfish is extremely rare in this region. Such database errors can profoundly impact ecological interpretations and fishery management, underscoring the urgent need for robust quality control measures in reference database curation.

Currently, quality control of reference databases in eDNA studies primarily relies on sequence filtering tools such as BLAST‐based similarity searches, primer matching, and taxonomic annotation validation. However, these approaches face notable limitations in practice (Schenekar et al. 2020). For example, although BLAST‐based searches allow for thresholds such as maximum e‐value and minimum identity, short eDNA fragments often lack sufficient interspecific variability and may exhibit substantial intraspecific overlap, leading to inaccurate taxonomic assignments (Claver et al. 2023; Curd et al. 2024). This limitation is intrinsic to the nature of short barcode sequences, rather than being solely due to database curation. Primer matching approaches are highly dependent on precise primer design and may miss valid sequences due to mismatches at primer‐binding sites. Allowing a limited number of mismatches during in silico screening can alleviate this issue to some extent and improve the detection of sequence variants (Jeunen et al. 2023). Annotation validation tools are often restricted to a single genetic region and rely on accurate original species identification, making them less effective for identifying complex issues such as hybridization, contamination, or cryptic speciation (Keck and Altermatt 2023; Meglecz 2023). As a result, these tools primarily function as preliminary filters rather than comprehensive quality control frameworks.

Phylogeny‐based strategies, which incorporate evolutionary relationships among sequences, offer a more effective means of detecting misclassifications and anomalies in reference databases compared to simpler sequence similarity methods. Existing tools such as *Meta‐Fish‐Lib* typically assess species monophyly to flag problematic entries, improving the reliability of fish reference databases (Collins et al. 2021). However, these existing approaches face several critical drawbacks. First, the construction of phylogenetic trees with all sequences available for large‐scale datasets is computationally demanding and impractical for thousands of sequences, greatly limiting scalability (Collins et al. 2021). Second, current methods generally rely heavily on manual curation without standardized or reproducible workflows, making the process subjective and potentially inconsistent. For example, some studies manually exclude suspicious sequences based on expert judgment or ad hoc taxonomic knowledge, without clearly defined criteria or scalable procedures (Jeunen et al. 2023; Keck and Altermatt 2023). This lack of structure limits reproducibility and increases the risk of overlooking subtle but systematic errors. Lastly, phylogeny‐based approaches inherently struggle with biological complexities such as gene flow, incomplete lineage sorting, and hybridization events, as these phenomena frequently violate monophyly assumptions and complicate the interpretation of tree topologies, potentially leading to misclassification or removal of authentic sequences (Redmond et al. 2023).

To overcome these limitations, we developed *PhyloRef*, which introduces three key improvements. First, by grouping sequences taxonomically prior to tree building and automating alignment and tree inference within a Snakemake workflow, *PhyloRef* improves computational scalability and enables large‐scale application across thousands of sequences. Second, by implementing structured anomaly classification and providing standardized visual outputs, *PhyloRef* reduces subjectivity, improves reproducibility, and minimizes reliance on ad hoc expert judgment. Third, instead of discarding sequences that fail monophyly, *PhyloRef* adopts a conservative strategy by flagging ambiguous cases with a “*similar_to=*” annotation (a label indicating uncertain phylogenetic placement), allowing users to recognize potential biological complexities, such as introgression or incomplete lineage sorting, without losing valuable evolutionary signal. Through these improvements, *PhyloRef* retains the advantages of phylogeny‐based validation while addressing the key limitations of existing phylogeny‐based approaches, including limited computational scalability, lack of standardized workflows, and difficulties in handling biological complexity.

In addition, we summarize the distribution of flagged (including “*similar_to=*” annotated) and removed records across taxa and data sources and briefly discuss whether these patterns may suggest taxon‐ or database‐specific issues. Patterns in these records may reflect a combination of database‐related issues (e.g., annotation inconsistencies, submission or formatting errors, contamination) and biological processes that can blur phylogenetic signal (e.g., introgression or incomplete lineage sorting). While PhyloRef does not attempt to distinguish these causes automatically, summarizing such patterns can help users prioritize manual verification and interpret downstream eDNA assignments more cautiously.

## 2 Materials and Methods

### 2.1 Workflow Overview and Implementation

**FIGURE 1:**
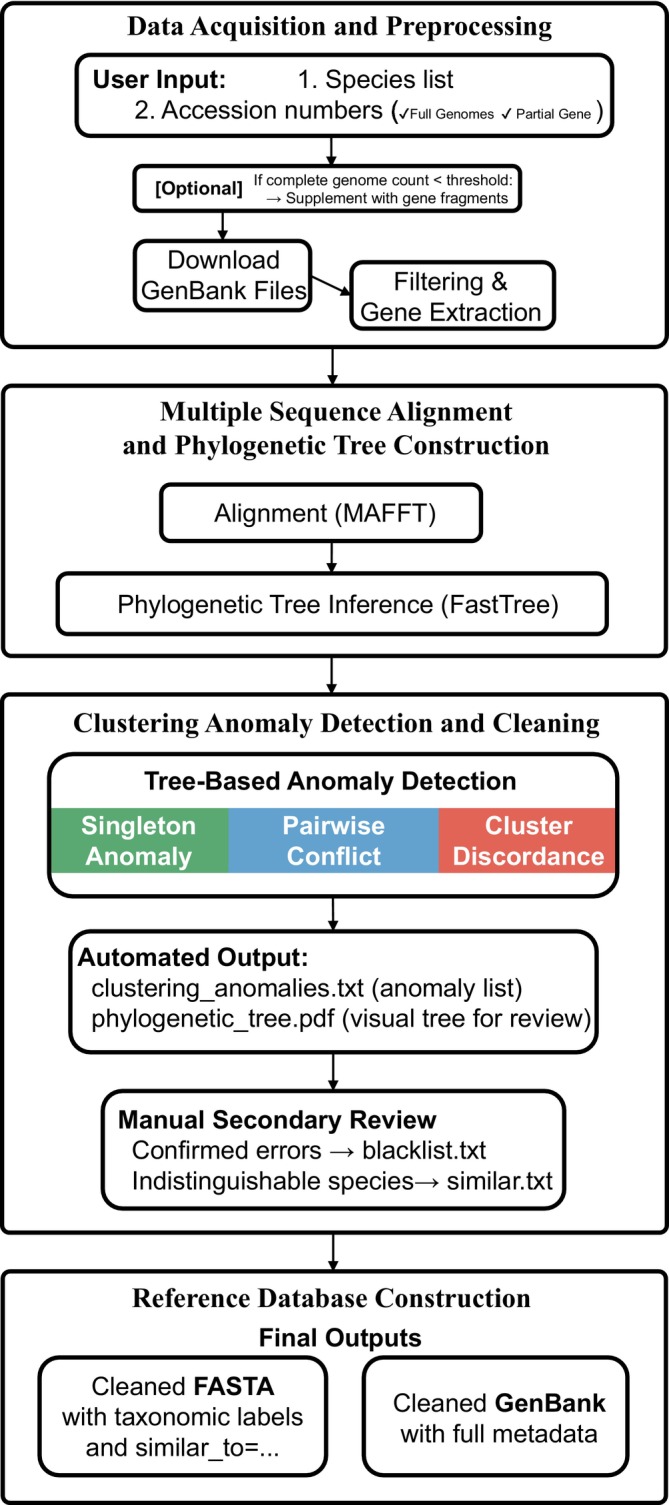
Overview of the *PhyloRef* pipeline. Schematic overview of the *PhyloRef* workflow, showing seven sequential modules. (1) Data acquisition: Bulk download of mitochondrial genomes from GenBank. (2) Preprocessing: Filtering by completeness, redundancy, and taxonomic annotation. (3) Gene extraction: Parsing protein‐coding and rRNA genes from GenBank files. (4) Grouping: Sequences clustered by order for computational scalability. (5) Alignment & tree building: MAFFT alignment and FastTree inference. (6) Anomaly detection: Classification of green, blue, and red anomalies based on phylogenetic placement. (7) Database construction: Removal of confirmed errors, annotation of ambiguous sequences, and compilation of curated reference libraries.

*PhyloRef* is a modular Snakemake (v7.32) (Koster and Rahmann 2012) workflow that accepts two types of input files: (1) a species name list or (2) a list of accession numbers (Figure 1). The species list option is typically used when users curate a reference database based on a checklist of target taxa or local biodiversity records. In contrast, the accession number option is well suited for cases where researchers extract data from NCBI using taxonomic identifiers (e.g., txid7777 for Chondrichthyes), which allows batch retrieval of all mitochondrial genomes or specific genes (e.g., COX1) within a clade. This input mode enables full reproducibility and precise control over downloaded datasets. The workflow consists of seven sequential modules: data acquisition, gene extraction, sequence alignment, phylogenetic tree construction, anomaly detection, manual curation, and final database assembly. Each module is implemented as an independent Snakemake rule, enabling reproducible execution on both local machines and high‐performance computing clusters. The full pipeline architecture is illustrated in Figure 1. Detailed documentation, example datasets, and usage tutorials are available on the GitHub repository: https://github.com/yannnnmai/PhyloRef. To facilitate reproducibility, the repository also provides a Conda environment specification file (environment.yaml), which we instantiate using Micromamba to manage software dependencies. Using this environment, the workflow can be executed via Snakemake, including the provided example dataset.

### 2.2 Data Acquisition and Preprocessing

#### 2.2.1 Automated Retrieval of Mitochondrial Sequences

*PhyloRef* supports two modes of data acquisition: retrieval based on species names or on accession numbers. Users must provide one of these files in plain‐text format, either a species list or an accession list. To specify outgroup sequences, it is highly recommended to include accession numbers of designated outgroup sequences in the first one or two lines, and define them under the *outgroup_ids* field in the configuration file (*config.yaml*). These accession numbers are automatically recognized as outgroups during tree rooting and anomaly detection, facilitating consistent rooting and accurate phylogenetic interpretation in subsequent analyses.

In species list mode, the workflow automatically constructs standardized Entrez queries for each target species (e.g., species name AND mitochondrion[filter] AND complete genome[title]) to retrieve its corresponding complete mitochondrial genome records. This mode is particularly suited for large‐scale database construction. In accession list mode, if users intend to incorporate specific gene fragments (e.g., 12S) into the reference database, they should prepare two separate accession lists, one for complete mitogenomes and another for the target gene region. These lists must be merged into a single file and deduplicated locally before running the workflow. Subsequent steps apply consistent filtering and subsampling procedures to all records, ensuring coherence and representativeness of the final database. This module outputs raw GenBank‐format files downloaded from NCBI, which are passed to downstream filtering and processing steps.

#### 2.2.2 Filtering and Species‐Level Subsampling

Sequence quality control is configured via the *config.yaml* file and consists of two phases: record‐level filtering and species‐level subsampling. In the record‐level filtering phase, users may select one of two mutually exclusive modes: The “*complete*” mode retains records annotated as “complete mitochondrial genome”, “whole mitochondrion”, or similar phrases in their GenBank definition lines, in order to capture a broader set of potentially complete mitogenomes. In practice, this filter performs a case‐insensitive partial match against the GenBank DEFINITION line, retaining records whose definitions contain any of these keywords (or close variants). This setting is optimized for applications that require full‐length genomes, supporting downstream multi‐gene concatenation and phylogenetic analysis (typically encompassing 13 protein‐coding genes plus 12S and 16S rRNA). The “*gene*” mode retains not only complete mitogenomes but also partial sequences containing user‐specified target genes (e.g., 12S, 16S, CYTB) that exceed a user‐defined length threshold (default: 400 bp). While this default value helps exclude extremely short or low‐quality fragments, users can adjust the threshold accordingly if conducting eDNA analyses with short barcode regions (e.g., < 200 bp, such as using the Teleo primers). To identify gene‐containing fragments, the workflow scans the *feature* annotations of each GenBank record and searches for gene names in the gene, product, and note fields using flexible regular expressions. For example, 12S may be recorded as “12S”, “12S rRNA”, “s‐rRNA”, or “small subunit ribosomal RNA”, while COI may appear as “COI”, “CO1”, “COX1”, or “cytochrome c oxidase subunit I”. If a target gene cannot be reliably extracted due to incomplete or inconsistent annotations, the entire sequence is retained to avoid unintentional data loss. In some cases, records may contain the 12S region but lack properly labeled features due to submission errors or legacy formatting. This mode is ideal for applications focused on single‐gene analysis or when complete genomes are scarce and fragmentary data must be maximally utilized. To ensure taxonomic accuracy, records containing ambiguous or non‐standard qualifiers such as “cf.”, “sp.”, “ssp.”, or “x” are excluded by default. However, users can override this behavior via specific parameters (e.g., *keep_cf: true*). In cases where such sequences are retained, their phylogenetic placement can be evaluated in later steps. Rather than being automatically discarded, some of these sequences may prove useful if they cluster consistently with confidently identified taxa. When handling RefSeq data, the system prioritizes representative records with an *NC_* accession prefix and removes redundant non‐RefSeq submissions with identical sequence content, ensuring that duplicate entries are discarded while all validated records are retained.

In the species‐level subsampling phase, the workflow aims to balance taxonomic representation and dataset size by retaining up to five sequences per species by default (adjustable via the *max_sequences*, default: 5). Only sequences that pass record‐level filtering, i.e., containing the target gene, meeting the minimum length threshold, and lacking ambiguous taxonomic qualifiers are considered for retention. To facilitate informed parameter tuning, the total number of available sequences per species before subsampling is logged and summarized in the output directory. In complete genome mode, identical sequences are removed and then, if five or fewer records are available for a given species, all are retained. If more than five exist, redundant entries are removed based on submitter identity and geographic sampling origin, with five representative sequences randomly kept. This heuristic approach, based on submitter identity and geographic sampling origin, may not fully capture intraspecific variation. While it helps reduce redundancy from oversampled studies, users should be aware that some genuine biological diversity may be lost. If preserving population‐level diversity is a priority, this filtering step can be disabled or adjusted accordingly. In the target gene mode, complete mitochondrial genomes are still prioritized. If fewer than five complete genome records are available for a given species, high‐quality gene fragments (i.e., sequences containing clearly annotated target genes, minimal ambiguous bases, and lengths exceeding the user‐defined threshold) are used to fill the remaining quota. During this process, redundant records are first removed based on sequence identity, submitter and geographic origin. The remaining sequences are then ranked by the length of the target gene region, with those containing the longest target gene sequences retained preferentially. The output of this module is a curated GenBank file containing subsampled sequences across species, which serves as input for downstream alignment and phylogenetic tree construction.

#### 2.2.3 Gene Extraction

Target gene extraction is based on feature annotations provided in GenBank files. Specifically, the workflow extracts the nucleotide sequences corresponding to user‐specified genes (e.g., 12S, CYTB, COI) by searching for gene names within the gene, product, and note fields of annotated features. Users can specify one or more mitochondrial gene regions via the configuration file. If no target genes are explicitly defined, the workflow will extract all annotated coding sequences (CDS) and rRNA genes by default, including the 13 standard protein‐coding genes (ND1–6, ND4L, COX1–3, ATP6, ATP8, and CYTB), as well as commonly used environmental DNA markers such as 12S and 16S rRNA. The extracted output consists of the full nucleotide sequences of the specified genes, ensuring that complete gene regions are retrieved for downstream analyses. To maximize compatibility with variable annotations, gene identification is performed using regular expression matching across the gene, product, and note fields, capturing common synonyms and naming variants (e.g., “COI”, “COX1”, “cytochrome c oxidase subunit I”).

Under the *filter. mode= gene* setting, it is recommended to maintain consistency in gene naming and extract only user‐defined target genes (e.g., 12S) to avoid inconsistencies across workflow steps. In contrast, when *filter. mode= complete*, the workflow allows users to specify multiple target genes for simultaneous extraction. The corresponding gene regions are retrieved from each complete mitogenome and concatenated into a single multi‐locus sequence, enabling the construction of alignment matrices for downstream phylogenetic analysis.

For records lacking complete annotations or missing target genes, the workflow retains the full sequence to ensure downstream data continuity and coverage. All extracted sequences are exported in three output formats to support diverse analysis goals: (1) Per‐gene FASTA files: A separate FASTA file is generated for each target gene, suitable for gene‐level species identification, primer evaluation, or simplified reference database construction. These files also support the addition of *similar_to=* tags to indicate phylogenetically indistinguishable but taxonomically distinct sequences. (2) Concatenated multi‐gene FASTA file (*concat.fa*): This file is used as the default input for multiple sequence alignment and phylogenetic tree construction. In gene mode, it is identical to the per‐gene FASTA files. In complete mode, the workflow extracts target gene regions from each mitogenome based on annotated coordinates and concatenates them following the standard gene order typically found in mitochondrial genomes. While these genes may not be physically adjacent in all species, they are joined in this canonical order to generate a composite sequence for multi‐locus phylogenetic analysis. If no target genes can be extracted for a given record, the full sequence is retained instead to preserve data integrity. (3) Full‐length sequence FASTA file: This includes the complete sequence of every retained record, regardless of gene extraction success. It serves both as the raw input for reference database construction and as the source file for sequence cleaning after clustering‐based anomaly detection. The *similar_to=* tags are primarily added based on this file.

In addition to GenBank‐derived records, users may incorporate their own FASTA sequences into the workflow. We recommend completing Steps 2.2.1–2.2.2 (data acquisition and preprocessing) before manually adding the formatted sequences to the FASTA files produced in the preprocessing directory. Each record should follow the PhyloRef header format: Genus_species|Accession_ID|RecordType|o__Order|f__Family|g__Genus where Accession_ID represents a unique identifier (e.g., an experimental or date‐based code) and RecordType indicates the source type (e.g., “Complete”, “Partial”, or “User_sequence”). For instance: Amblyraja_georgiana|LOCAL_2025_001|User_sequence|o__Rajiformes|f__Rajidae|g__Amblyraja This standardized naming scheme facilitates consistent parsing and accurate taxonomic grouping. Once added, the workflow can be resumed from the grouping and alignment stage (§ 2.3), allowing user‐generated sequences to be integrated seamlessly into downstream analyses and final database construction.

### 2.3 Multiple Sequence Alignment and Phylogenetic Analysis

To construct a robust phylogenetic framework, *PhyloRef* groups the concatenated sequences (from *concat.fa*) by taxonomic rank prior to alignment and tree building. By default, sequences are grouped by their order‐level classification, with a maximum threshold of 2000 sequences per file (configurable via *max_per_file*). Orders are first sorted in descending order based on their sequence counts. The remaining orders are then sequentially combined into groups, ensuring each group's total sequence count does not surpass the set threshold. If an individual order exceeds this threshold, it is automatically placed into its own group without further combination. Each resulting group is output as a separate file for phylogenetic reconstruction to increase tractability. Users may also specify one or more outgroup sequences via the *outgroup_ids* parameter. These sequences are forcibly retained in all subsets to ensure consistent rooting of the resulting trees.

Each subset data undergo multiple sequence alignment using MAFFT v7.520 (Katoh and Standley 2013), with customizable execution path and number of threads (30 threads were used in this study). The resulting alignments are then passed to FastTree v2.1.10 (Price et al. 2010) to infer maximum likelihood phylogenies. The tree construction is performed with the *‐nt* and *‐fastest* options enabled to accelerate nucleotide‐based topology reconstruction, making the process suitable for large‐scale datasets. This step outputs both the alignment files (.*aln*) and corresponding phylogenetic trees (.*nwk*) for each subset, which serve as the foundation for downstream anomaly detection and database curation.

### 2.4 Phylogenetic Anomaly Detection and Manual Review

#### 2.4.1 Automated Detection and Classification of Anomalies

After constructing order‐level phylogenetic trees, *PhyloRef* performs automated topological anomaly detection for each subtree to identify potential misannotations or phylogenetic inconsistencies. The classification is based on the consistency between phylogenetic topology and taxonomic annotations. The workflow first groups sequences by species and classifies them into three categories based on their count: species with only one sequence, with two sequences, and with three or more sequences. Each category is then evaluated using a specific set of tree‐based criteria to detect phylogenetic anomalies. Based on this, flagged sequences are assigned to one of three types: Type I (Singleton anomaly‐green): A species represented by a single sequence that clusters far from its expected genus‐, family‐, or order‐level clade. Such outliers are likely due to annotation errors or isolated misentries. Type II (Pairwise conflict‐blue): A species with two sequences that fail to cluster together. If one sequence clusters with other members of the same genus, family, or order while the other does not, the latter is flagged as an anomaly (blue). If both sequences are interspersed with other species and exhibit unclear clustering patterns, both are flagged, indicating potential taxonomic ambiguity. Type III (Cluster discordance‐red): A species represented by three or more sequences, with at least one sequence clearly diverging from the main clade. This may reflect intraspecific divergence, data contamination, or poorly defined species boundaries. All detected anomalies are recorded in a *clustering_anomalies.txt* file. Corresponding rooted phylogenetic trees are rendered as PDF files, using the user‐defined outgroup sequences specified in the configuration file to aid in manual inspection.

#### 2.4.2 Manual Review and Tag Assignment

Users can manually review all flagged sequences based on the PDF tree and anomaly types, and curate the *clustering_anomalies.txt* file accordingly. During this process, sequences should be visually inspected according to the color‐coded markers in the PDF tree and evaluated in conjunction with their taxonomic information. To improve transparency and consistency, manual review follows a predefined checklist focusing on (i) concordance between the taxonomic label and tree placement, (ii) inspection of GenBank metadata (e.g., DEFINITION and source qualifiers), (iii) basic sequence‐level sanity checks (e.g., unexpected length or excessive ambiguous bases), and (iv) confirmation of expected phylogenetic affinity based on taxonomic resources and relevant literature for the focal group. The specific operations are as follows: if a sequence is confirmed to be erroneous, its corresponding entry in the *clustering_anomalies.txt* file should remain unchanged; if the sequence is determined to be correct (a false positive), the corresponding entry should be removed from the *clustering_anomalies.txt* file; if sequences cannot be distinguished on the phylogenetic tree, their entries should be deleted from the *clustering_anomalies.txt* file, and their accession numbers should be recorded in the *similar.txt* file, with each line representing one group of indistinguishable sequences. The results of this manual review directly affect the accuracy of subsequent database cleaning and annotation standardization. These indistinguishable groups recorded in similar.txt are subsequently used to generate the *similar_to=* annotations in the curated FASTA outputs.

### 2.5 Database Construction and Version Control

Following anomaly detection and gene extraction, the finalized database is compiled by consolidating all retained sequences and applying standardized annotations. Clean and blacklisted records are organized into separate directories, with accompanying summary files (e.g., *cleanlist.txt*, *blacklist.txt*) documenting filtering outcomes for traceability.

To accommodate various downstream applications, the database is exported in both GenBank and FASTA formats. GenBank files preserve all original annotation fields. In the curated FASTA outputs, we append a *similar_to=* tag to the sequence header when it belongs to an indistinguishable group curated in similar.txt. Each line in similar.txt represents an accession group that is indistinguishable on the phylogenetic tree. When writing the FASTA header, we summarize this group at the species level by retaining a single representative accession per species, prioritizing RefSeq accessions (i.e., those starting with “NC_”). If no “NC_” record is available for a species, we select the first accession in alphanumeric (lexicographic) order among the retained records for that species. The *similar_to=* field lists these representative accessions as a comma‐separated set. For each retained record, *similar_to=* summarizes the representative accessions from its corresponding indistinguishable group; therefore, multiple accessions appearing in *similar_to=* indicate that this sequence cannot be reliably distinguished from multiple species in subsequent taxonomic assignment, given the sequence markers used in this study and the current dataset coverage. For example: Microphysogobio_linghensis|NC_086468.1|Concat|o__Cypriniformes|f__Gobionidae|g__Microphysogobio|similar_to= NC_051965.1, NC_086467.1|. Here, NC_051965.1 corresponds to *Microphysogobio tungtingensis* and NC_086467.1 corresponds to *Microphysogobio microstomus* , indicating that the *M. linghensis* record above cannot be reliably separated from these species under the current marker set and dataset coverage.

Each release is versioned using the format MitoDB_vYYMMDD and archived with a summary report that includes sequence counts, taxonomic coverage, and processing statistics. This structure supports reproducibility, long‐term maintenance, and comparative analyses across database versions.

### 2.6 Case Study: Mitogenomic Database Construction for Ray‐Finned and Cartilaginous Fishes

As a proof of concept, we applied *PhyloRef* to two major vertebrate lineages: ray‐finned fishes (Actinopterygii) and cartilaginous fishes (Chondrichthyes). These two groups were selected as representative test cases because they exhibit broad taxonomic diversity, ecological importance across both marine and freshwater environments, and extensive coverage in publicly available sequence repositories. Specifically, we included all complete mitochondrial genome sequences available for these two classes in NCBI (as of February 2025), ensuring comprehensive taxonomic representation and enabling a robust validation of PhyloRef's anomaly‐detection capabilities.

All retrieved mitogenomic records were processed through the PhyloRef workflow, which encompasses sequence filtering, anomaly detection, and database curation. This case study was designed to evaluate the workflow's scalability, accuracy, and cross‐taxonomic applicability under highly divergent evolutionary contexts. Detailed retrieval statistics, filtering results, and anomaly‐detection outcomes are presented in the Results section. All curated datasets and intermediate outputs generated in this case study have been publicly deposited in Zenodo (DOI: https://doi.org/10.5281/zenodo.17285318).

## 3 Results

### 3.1 Data Acquisition and Initial Filtering

The analyses presented here are based exclusively on complete mitochondrial genome datasets, which provide the most complete basis for applying all mitochondrial eDNA markers and more reliable large‐scale phylogenetic validation. Although partial gene fragments (e.g., COX1 in Chondrichthyes and 12S in Actinopterygii) were not included in this analysis, the workflow is fully compatible with fragmentary datasets, and step‐by‐step guidance for such applications is provided in the GitHub documentation (see Data Availability).

Bulk retrieval from GenBank yielded 2234 chondrichthyan accessions (retrieved on February 13, 2025) and 16,795 Actinopterygian accessions (retrieved on February 26, 2025). After applying the filtering criteria described above—including the removal of incomplete entries, hybrids, and records annotated with “cf.” or “sp.” and retaining a maximum of five representative sequences per species—the curated chondrichthyan dataset comprised 389 sequences spanning 269 species. With the addition of two outgroup sequences, the final dataset contained 391 records. For Actinopterygii, 7570 sequences representing 4957 species were retained, yielding a final total of 7572 with two outgroups included.

### 3.2 Phylogenetic Analysis and Anomaly Detection

The resulting tree topology showed that most species clustered clearly and consistently at the order or family level. However, a subset of sequences exhibited marked clustering deviations, which served as critical references for downstream anomaly detection (File S1).

**FIGURE 2:**
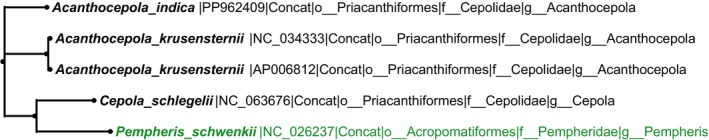
Type I (green) anomaly in *Pempheris schwenkii* : One sequence (NC_026237, highlighted in green) clusters within Cepolidae instead of grouping with its conspecifics in Pempheridae, indicating a misplacement.

**FIGURE 3:**
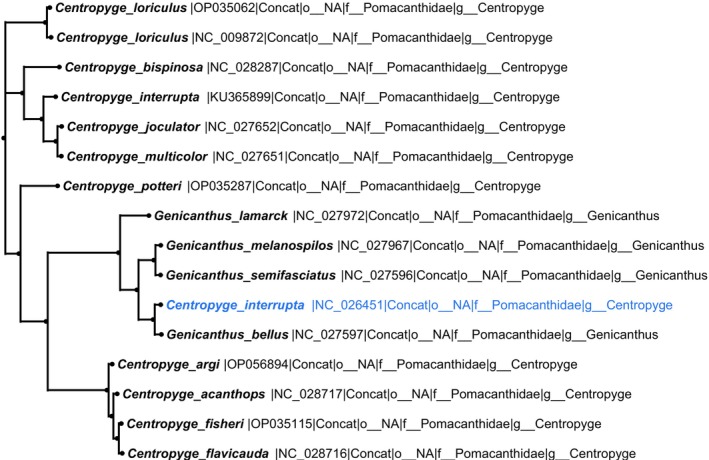
Type II (blue) anomaly in *Centropyge interrupta*: One sequence (NC_026451, highlighted in blue) clusters with Genicanthus species instead of grouping with its conspecific sequence (KU365899), demonstrating an incongruent phylogenetic placement.

**FIGURE 4:**
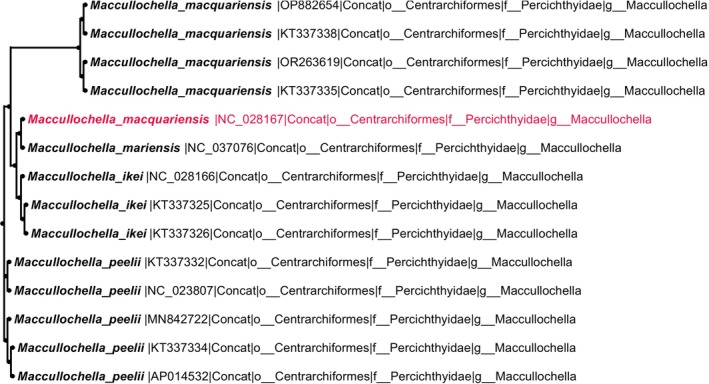
Type III (red) anomaly in *Maccullochella macquariensis* : One RefSeq sequence (NC_028167, highlighted in red) shows discordant clustering relative to other conspecific sequences, representing a multi‐sequence inconsistency within the species‐level clade.

Detected anomalies were classified into three categories based on their clustering patterns within phylogenetic trees (Figures 2, 3, 4). For example, a Type I anomaly involved sequence NC_026237 ( *Pempheris schwenkii* ), which clustered separately from other Pempheridae species (Figure 2). A representative Type II anomaly was NC_026451 (*Centropyge interrupta*), which incorrectly clustered with *Genicanthus* rather than with its conspecific sequence KU365899 (Figure 3). Lastly, Type III anomalies were exemplified by NC_028167 ( *Maccullochella macquariensis* ), which displayed aberrant clustering despite being a RefSeq entry (Figure 4). Although RefSeq records undergo additional curation compared to other entries in GenBank, they are not fully error‐free. Previous studies have identified erroneous entries in RefSeq that have not yet been removed from the database(Goudey et al. 2022).

For the chondrichthyan dataset, *PhyloRef* flagged seven Type I (green) anomalies. Manual inspection confirmed two as genuine phylogenetically inconsistent sequences that were removed, while five were retained due to insufficient phylogenetic resolution to justify exclusion. Ten Type II (blue) anomalies were detected, with four excluded after validation and six retained, and three Type III (red) anomalies were identified, all of which were excluded. In total, nine anomalous sequences were removed from the chondrichthyan dataset.

In Actinopterygii, 102 green anomalies were identified, of which 75 (73.5%) were validated as misassigned sequences and excluded, while 27 were retained because they maintained appropriate order‐level placement despite unresolved genus‐ or family‐level assignment. A total of 363 blue anomalies were identified, with 78 (21.5%) excluded as confirmed inconsistencies and 285 retained. Finally, 317 red anomalies were detected; 248 were removed as demonstrably discordant, while 69 were retained for further study due to persistent topological uncertainty despite systematic evaluation. In total, 401 anomalous sequences were excluded from the Actinopterygian dataset.

Notably, *PhyloRef* also flagged inconsistencies in RefSeq (NC_) entries (O'Leary et al. 2016; Schenekar et al. 2020). Among 3788 Actinopterygian NC_ sequences, 128 (3.38%) were removed, while 6 out of 229 (2.62%) chondrichthyan NC_ sequences were excluded due to phylogenetic conflicts. These findings highlight that metadata‐driven validation alone is insufficient, underscoring the value of integrating topology‐based methods such as *PhyloRef* into reference database construction.

**FIGURE 5:**
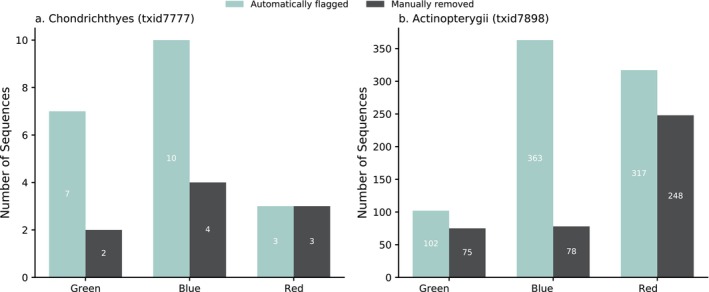
Phylogenetic anomaly detection outcomes: Automated flagging versus manual curation. Bars show the number of phylogenetic anomalies detected by PhyloRef in (a) Chondrichthyes (txid7777) and (b) Actinopterygii (txid7898). Green, blue, and red categories correspond respectively to Type I (singleton anomaly), Type II (pairwise conflict), and Type III (cluster discordance). Green bars represent sequences automatically flagged by PhyloRef, while gray bars indicate those manually confirmed and removed after visual inspection of phylogenetic trees.

The comparison between automated and manually confirmed anomalies is summarized in Figure 5. Across both datasets, most anomalies detected by PhyloRef were confirmed upon manual inspection, demonstrating good agreement between automated and manual evaluation. Type III (red) anomalies showed the highest consistency between automated and manual results, indicating strong reliability of PhyloRef's cluster‐level detection. Type I (green) anomalies were also frequently validated, though with moderate variation between taxa. In contrast, Type II (blue) anomalies displayed the largest discrepancy, suggesting that many of these pairwise conflicts reflect genuine phylogenetic uncertainty among closely related species rather than clear misannotation. Beyond these detection statistics, we next evaluated how annotation quality and potential taxonomic inconsistencies were distributed across major fish families to identify clade‐specific patterns of error and ambiguity.

### 3.3 Taxonomic Distribution and Quality Assessment

**FIGURE 6:**
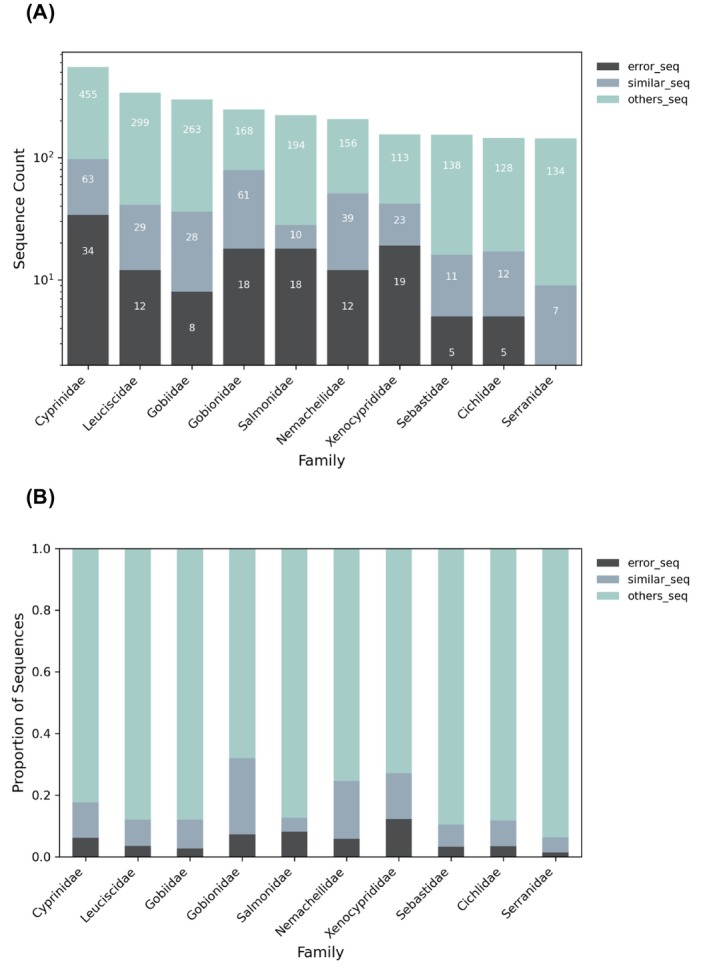
Annotation quality assessment across the top 10 Actinopterygian families. (a) Log‐scaled sequence counts categorized into error_seq (confirmed erroneous sequences), similar_seq (phylogenetically ambiguous sequences flagged with similar_to), and others_seq (all remaining sequences). (b) Proportional distribution of the same categories normalized to 1.0 per family.

As part of the reference database evaluation, we assessed the taxonomic distribution and annotation quality of curated mitochondrial sequences across the top 10 most represented families of Actinopterygii (Figure 6). The results are summarized using two complementary stacked bar charts: first, a stacked bar chart displays log‐transformed absolute sequence counts per family, categorized as either erroneous sequences (error_seq), ambiguous/indistinguishable sequences (similar_seq), or reliable sequences (others_seq), with exact counts numerically labeled within each bar. Second, a proportional stacked bar chart normalizes these categories to a total of 1.0 per family, emphasizing taxonomic groups where problematic annotations (error_seq and similar_seq) represent an unusually high proportion of sequences. Together, these visualizations enable simultaneous evaluation of both the magnitude and relative severity of annotation issues across major fish lineages.

Our analyses revealed considerable variation in sequence quantity and quality across families, with total sequence counts ranging from 143 to 552 per family. The proportion of confirmed erroneous sequences (error_seq) showed a 1.40%–12.26% range, while ambiguous sequences (similar_seq) accounted for 4.50%–24.70% at family level. Notably, while Cyprinidae possessed the largest absolute sequence count (> 500), families such as Gobionidae, Xenocyprididae, and Nemacheilidae exhibited substantially higher proportions of problematic annotations—reaching approximately 32%, 27%, and 25%, respectively. These results indicate that the proportion of problematic records (error_seq and similar_seq) can vary markedly among families, including those with only moderate total sequence counts. Accordingly, family‐level summaries of error_seq and similar_seq provide a practical overview of where reference records may warrant closer scrutiny in downstream eDNA analyses (Weigand et al. 2019).

Additionally, sequences that exhibited ambiguous phylogenetic placements without definitive evidence of error were retained in the final database but explicitly annotated with a “*similar_to=*” label. This included 9 sequences from Chondrichthyes and 597 from Actinopterygii, thereby indicating potential taxonomic uncertainty and supporting conservative downstream interpretation in subsequent eDNA and phylogenetic analyses (Table S1).

### 3.4 Ambiguous Phylogenetic Placements

**FIGURE 7:**
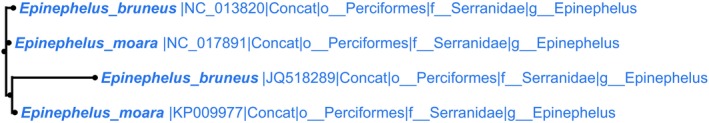
Phylogenetic ambiguity between *Epinephelus bruneus* and *E. moara*. Recently diverged sister species show nearly identical mitochondrial genomes, preventing clear phylogenetic separation.

**FIGURE 8:**
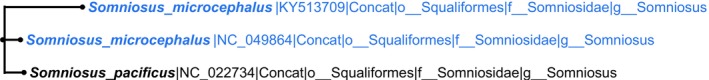
Phylogenetic ambiguity between *Somniosus microcephalus* and *S. pacificus* . Mitochondrial haplotypes overlap, indicating unresolved species boundaries.

**FIGURE 9:**
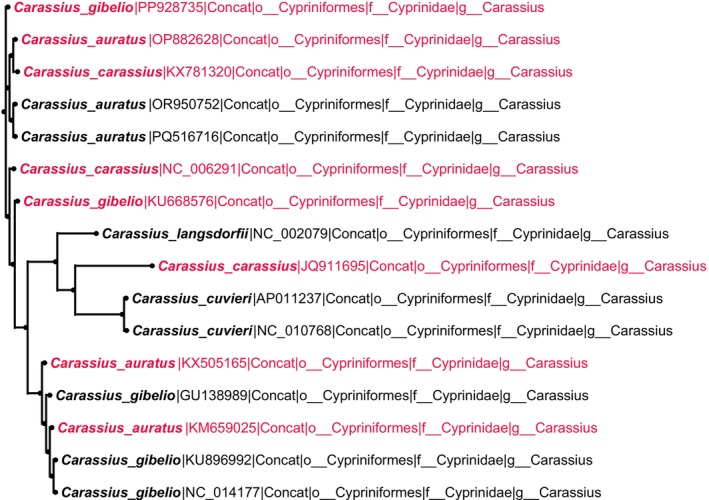
Phylogenetic ambiguity within Carassius species. Mitochondrial sequences of multiple species show extensive overlap, indicating limited differentiation.

In addition to clearly misassigned sequences, our analyses also revealed phylogenetically ambiguous cases where mitochondrial genomes showed insufficient resolution to distinguish closely related taxa. Three representative patterns were observed. First, recently diverged sister species such as *Epinephelus bruneus* (NC_013820, JQ518289) and *E. moara* (NC_017891, KP009977) exhibited near‐identical mitochondrial sequences, resulting in unresolved clustering (Figure 7). Second, overlapping placements were observed in *Somniosus microcephalus* (KY513709, NC_049864) and *S. pacificus* (NC_022734), which did not form distinct monophyletic groups (Figure 8). Third, members of the genus *Carassius* ( *C. carassius* , *C. cuvieri* , *C. langsdorfii* , *C. auratus* , and *C. gibelio* ) showed minimal differentiation and broad topological overlap, consistent with a recent radiation (Figure 9). These ambiguous cases were not treated as errors but retained with explicit similar_to annotations for downstream applications.

### 3.5 Final Database Construction

The curated database comprises 380 high‐quality mitochondrial genomes representing 266 chondrichthyan species and 7258 genomes from 4887 actinopterygian species (excluding outgroups). These sequences were retained following standardized filtering, alignment, and anomaly removal steps. The database is provided in two complementary formats to accommodate different analytical purposes. The GenBank (GB) version preserves full annotation metadata, including references, genomic features, and gene boundaries, making it suitable for detailed molecular and comparative studies. In contrast, the FASTA (FA) version offers streamlined sequence data with standardized taxonomic labels (order, family, and genus), facilitating rapid comparative analyses, visualization, and phylogenetic applications. All curated sequences and corresponding phylogenetic trees are publicly accessible on Zenodo (DOI: https://doi.org/10.5281/zenodo.17285318). By removing confirmed anomalous references and flagging phylogenetically ambiguous records with “*similar_to=*”, the curated outputs provide a more conservative basis for downstream eDNA taxonomic assignment by alerting users to residual phylogenetic ambiguity.

## 4 Discussion

### 4.1 Management of Phylogenetically Ambiguous Sequences

Beyond clearly identifiable anomalies, our analysis revealed cases where mitochondrial genomes showed insufficient differentiation to reliably distinguish certain closely related species or groups. Such ambiguous placements, often resulting from recent speciation, mitochondrial introgression, or rapid evolutionary radiation, reflect intrinsic limitations of mitochondrial markers rather than database errors. To address this, sequences were retained but explicitly flagged with similar_to annotations. This approach ensures transparency and enables accurate interpretation in downstream analyses (Figures 7, 8, 9).

Our analysis identified cases where recently diverged sister species showed minimal mitochondrial differentiation, as exemplified by *Epinephelus bruneus* (NC_013820, JQ518289) and *E. moara* (NC_017891, KP009977) (Figure 7). These taxa exhibited near‐identical nucleotide profiles that precluded clear phylogenetic separation, likely reflecting insufficient evolutionary time for accumulation of diagnostic mitochondrial mutations since their speciation event (Amorim et al. 2021).

Our analysis also revealed a distinct category of phylogenetically ambiguous sequences resulting from historical introgression events or incomplete speciation processes, where persistent gene flow obscures clear species boundaries. A representative case involves the mitochondrial sequences of *Somniosus microcephalus* (KY513709, NC_049864) and *S. pacificus* (NC_022734), which showed overlapping clustering patterns without forming distinct monophyletic groups. These findings, visually documented in Figure 8, are consistent with either mitochondrial introgression following secondary contact or incomplete establishment of reproductive barriers during early divergence phases. Such patterns challenge conventional species delimitation approaches based solely on mitochondrial data and highlight the need for complementary nuclear markers to accurately reconstruct complex evolutionary histories involving hybridization or nascent speciation events (Timm et al. 2023).

The genus *Carassius* exemplifies a third major category of phylogenetic complexity, where rapid evolutionary radiation has resulted in minimal mitochondrial differentiation among descendant species. Our analysis of *C. carassius* , *C. cuvieri* , *C. langsdorfii* , *C. auratus* , and *C. gibelio* revealed extensively overlapping clustering patterns (Figure 9), consistent with a recent and explosive diversification event (Gu et al. 2022). Such phylogenetic blurring, characteristic of adaptive radiations across multiple taxa, poses significant challenges for species delineation using mitochondrial markers alone. These findings underscore the necessity of incorporating nuclear genomic data and morphological evidence when working with rapidly radiating groups, where conventional molecular barcoding approaches often prove inadequate for resolving complex evolutionary relationships.

To address cases of indistinguishable sequences identified through mitochondrial analysis, *PhyloRef* employs a conservative annotation strategy by marking these sequences with a *similar_to=* tag in FASTA headers while retaining representative entries. This approach yields multiple scientific benefits. First, it preserves biologically significant variation that may reflect important evolutionary processes such as ongoing speciation, introgressive hybridization, or recent adaptive radiation, thereby preventing the loss of valuable phylogenetic signal that might occur with outright removal. Second, the explicit annotation serves as a critical warning for downstream applications, particularly in sensitive environmental DNA (eDNA) metabarcoding studies, prompting researchers to incorporate supporting evidence from nuclear genomic data or morphological characteristics when definitive taxonomic assignment is required. This methodological framework proves particularly valuable for analyzing complex environmental samples, where it significantly reduces false positive identifications while maintaining comprehensive sequence representation, ultimately strengthening the reliability of biodiversity assessments in ecological monitoring and conservation planning.

These findings underscore the limitations of relying solely on database curation status and highlight the importance of incorporating topology‐based validation into routine reference library construction workflows. Furthermore, some retained anomalies, particularly those showing minor divergence or ambiguous placements, may represent genuine biological phenomena including mitochondrial introgression, incomplete lineage sorting, or undetected cryptic speciation. Such cases highlight the need for follow‐up studies incorporating nuclear genetic markers, morphological characterization, and expanded geographic sampling to enhance taxonomic resolution and improve reference database quality. This highlights the need to move beyond purely metadata‐based assessments and embrace integrated phylogenetic validation (Toews and Brelsford 2012).

### 4.2 Strengths and Current Limitations of *PhyloRef*

*PhyloRef* addresses critical challenges in reference database curation by integrating phylogeny‐based validation into a reproducible workflow. Its main strength lies in leveraging full mitochondrial genomes (13 protein‐coding and 2 rRNA genes), which improves phylogenetic resolution compared to traditional single‐marker approaches such as COI or 12S (Brys et al. 2023). Another key innovation is the structured anomaly classification (Types I–III) together with the similar_to annotation, which enables systematic handling of both obvious errors and biologically ambiguous cases. This dual strategy helps improve transparency and reduces subjective manual curation, a limitation in earlier frameworks.

Compared with existing tools, *PhyloRef* provides a complementary workflow‐level solution for reference‐library curation. Sequence‐similarity methods (e.g., BLAST‐based pipelines) allow rapid screening but often fail to detect misassignments among closely related taxa (Claver et al. 2023; Curd et al. 2024). Primer‐based pipelines such as *CRABS* (Jeunen et al. 2023) are efficient for targeted recovery but limited by primer specificity. Taxonomy‐dependent systems, including *refdb* and *COInr* (Keck and Altermatt 2023; Meglecz 2023), may propagate pre‐existing annotation errors due to their reliance on metadata accuracy. Phylogeny‐aware frameworks such as *Meta‐Fish‐Lib* (Collins et al. 2021) improve detection of non‐monophyletic taxa but generally require extensive manual work and face scalability issues with large datasets. Related tree‐based approaches such as *SATIVA* (Kozlov et al. 2016) use phylogenetic placement on a reference tree to detect putative taxonomic mislabels; however, their performance depends on the quality and taxonomic representativeness of the underlying reference alignment and tree, and they can be computationally demanding for very large datasets. By contrast, *PhyloRef* combines automated anomaly detection with manual visualization support, providing both efficiency and precision. In addition, PhyloRef adopts a divide‐and‐conquer strategy by partitioning sequences into taxonomic groups before alignment and tree inference, which allows multiple trees to be computed in parallel and avoids the cost of constructing a single, extremely large phylogeny. This design improves scalability for large reference libraries. Moreover, instead of removing all non‐monophyletic cases, PhyloRef retains ambiguous records and annotates them with a *similar_to=* tag, thereby providing a conservative reference set while explicitly signaling uncertainty to downstream eDNA analyses.

Nevertheless, several limitations remain. The current focus on mitochondrial genomes restricts detection of discordances arising from nuclear introgression or incomplete lineage sorting (Breton and Stewart 2015). Sparse sampling in certain groups (e.g., deep‐sea chondrichthyans) reduces inference reliability, while the short amplicons typical of eDNA metabarcoding (~200–300 bp) may yield ambiguous phylogenetic signal, potentially inflating anomaly detection (Hending 2025). Finally, although *PhyloRef* reduces manual effort, expert review is still required to interpret ambiguous cases, particularly those involving hybridization or cryptic species complexes. Taken together, *PhyloRef* provides a scalable and transparent solution that complements existing curation methods, supporting more reliable applications ranging from eDNA metabarcoding to evolutionary studies.

### 4.3 Future Development Priorities

While *PhyloRef* successfully overcomes numerous challenges in reference database construction, we identify four critical areas for future development to enhance its scalability, accuracy, and versatility. First, implementing machine‐learning models (e.g., random forests or deep‐learning classifiers) may improve anomaly‐detection performance. These advanced analytical approaches would minimize manual verification requirements while better identifying complex evolutionary patterns like hybridization events, genetic introgression, and rapid radiation scenarios that often elude conventional phylogenetic methods (Cordier et al. 2019; Keck, Brantschen, and Altermatt 2023; Keck, Couton, and Altermatt 2023).

Second, expanding beyond mitochondrial markers represents a crucial advancement. Incorporating nuclear genomic loci (e.g., ITS, 18S, and other conserved nuclear genes) would address current limitations in resolving intricate evolutionary histories involving gene flow or hybridization. This multi‐marker framework would provide substantially improved taxonomic resolution for closely related species and introgressed lineages (Dietz et al. 2022). Third, developing an automated updating system would ensure long‐term database relevance. This module would systematically retrieve new sequences from public repositories (e.g., NCBI), apply quality control protocols, and maintain version control to support data traceability. Such continuous integration would maintain database currency amidst the exponential growth of genomic data while standardizing data management practices. Finally, addressing computational constraints through algorithm optimization for parallel processing and distributed computing architectures would further enhance performance for large‐scale datasets. Coupled with these improvements, an intuitive web interface would increase accessibility for non‐specialists, streamlining submission, customization, and interpretation.

## 5 Conclusion

We introduce *PhyloRef*, a semi‐automated workflow for reference database curation that integrates phylogenetic topology analysis into a reproducible Snakemake framework. By leveraging complete mitochondrial genomes and supporting structured anomaly detection and similarity‐based annotation, *PhyloRef* improves the reliability of sequence data used in environmental DNA (eDNA) studies. Validation on datasets from Chondrichthyes and Actinopterygii demonstrates its capacity to detect misannotations, ambiguous placements, and potential biological signals such as introgression or incomplete lineage sorting. Overall, *PhyloRef* offers a practical and scalable tool for improving reference sequence quality, helping researchers reduce annotation errors and interpret taxonomic uncertainties with greater confidence.

## Author Contributions

**Yan Mai:** conceptualization (equal), data curation (equal), formal analysis (equal), investigation (equal), validation (equal), writing – original draft (equal). **Chenhong Li:** conceptualization (equal), funding acquisition (equal), resources (equal), writing – review and editing (equal).

## Funding

This research was supported by the National Key Research and Development Program of China (2022YFC2601301) and the Shanghai Science and Technology Commission (no. 23010502500, Capacity building project of local colleges and universities).

## Conflicts of Interest

The authors declare no conflicts of interest.

## Supporting information


**Table S1:** Genera and representative species showing ambiguous phylogenetic placements detected by PhyloRef. For each genus, one representative species and its ambiguous congeners are listed.

## Data Availability

All workflow scripts, configuration files, and documentation are openly available at https://github.com/yannnnmai/PhyloRef. The curated mitochondrial genome database in FASTA and GenBank formats, together with the full phylogenetic trees annotated and visually highlighted according to anomaly detection results, are archived on Zenodo under DOI: https://doi.org/10.5281/zenodo.17285318.

## References

[ece373159-bib-0001] Amorim, K. D. J. , G. W. W. F. D. Costa , M. B. Cioffi , A. Tanomtong , L. A. C. Bertollo , and W. F. Molina . 2021. “A New View on the Scenario of Karyotypic Stasis in Epinephelidae Fish: Cytogenetic, Historical, and Biogeographic Approaches.” Genetics and Molecular Biology 44, no. 4: e20210122. 34807969 10.1590/1678-4685-GMB-2021-0122 PMC8608104

[ece373159-bib-0002] Breton, S. , and D. T. Stewart . 2015. “Atypical Mitochondrial Inheritance Patterns in Eukaryotes.” Genome 58, no. 10: 423–431. 26501689 10.1139/gen-2015-0090

[ece373159-bib-0003] Brys, R. , D. Halfmaerten , T. Everts , C. Van Driessche , and S. Neyrinck . 2023. “Combining Multiple Markers Significantly Increases the Sensitivity and Precision of eDNA‐Based Single‐Species Analyses.” Environmental DNA 5, no. 5: 1065–1077.

[ece373159-bib-0004] Claver, C. , O. Canals , L. G. de Amézaga , I. Mendibil , and N. Rodriguez‐Ezpeleta . 2023. “An Automated Workflow to Assess Completeness and Curate GenBank for Environmental DNA Metabarcoding: The Marine Fish Assemblage as Case Study.” Environmental DNA 5, no. 4: 634–647.

[ece373159-bib-0005] Collins, R. A. , G. Trauzzi , K. M. Maltby , et al. 2021. “Meta‐Fish‐Lib: A Generalised, Dynamic DNA Reference Library Pipeline for Metabarcoding of Fishes.” Journal of Fish Biology 99, no. 4: 1446–1454. 34269417 10.1111/jfb.14852

[ece373159-bib-0006] Cordier, T. , A. Lanzén , L. Apothéloz‐Perret‐Gentil , T. Stoeck , and J. Pawlowski . 2019. “Embracing Environmental Genomics and Machine Learning for Routine Biomonitoring.” Trends in Microbiology 27, no. 5: 387–397. 30554770 10.1016/j.tim.2018.10.012

[ece373159-bib-0007] Curd, E. E. , L. Gal , R. Gallego , K. Silliman , S. Nielsen , and Z. Gold . 2024. “rCRUX: A Rapid and Versatile Tool for Generating Metabarcoding Reference Libraries in R.” Environmental DNA 6, no. 1: e489. 38370872 10.1002/edn3.489 PMC10871694

[ece373159-bib-0008] Dietz, L. , J. Eberle , C. Mayer , et al. 2022. “Standardized Nuclear Markers Improve and Homogenize Species Delimitation in Metazoa.” Methods in Ecology and Evolution 14, no. 2: 543–555.

[ece373159-bib-0009] Duhamet, A. , C. Albouy , V. Marques , S. Manel , and D. Mouillot . 2023. “The Global Depth Range of Marine Fishes and Their Genetic Coverage for Environmental DNA Metabarcoding.” Ecology and Evolution 13, no. 1: e9672. 36699576 10.1002/ece3.9672 PMC9846838

[ece373159-bib-0010] Dziedzic, E. , B. Sidlauskas , R. Cronn , et al. 2023. “Creating, Curating and Evaluating a Mitogenomic Reference Database to Improve Regional Species Identification Using Environmental DNA.” Molecular Ecology Resources 23, no. 8: 1880–1904. 37602732 10.1111/1755-0998.13855

[ece373159-bib-0011] Goudey, B. , N. Geard , K. Verspoor , and J. Zobel . 2022. “Propagation, Detection and Correction of Errors Using the Sequence Database Network.” Briefings in Bioinformatics 23, no. 6: bbac416. 36266246 10.1093/bib/bbac416 PMC9677457

[ece373159-bib-0012] Gu, Q. , S. Wang , H. Zhong , et al. 2022. “Phylogeographic Relationships and the Evolutionary History of the *Carassius auratus* Complex With a Newly Born Homodiploid Raw Fish (2nNCRC).” BMC Genomics 23, no. 1: 242. 35350975 10.1186/s12864-022-08468-x PMC8962218

[ece373159-bib-0013] Hending, D. 2025. “Cryptic Species Conservation: A Review.” Biological Reviews of the Cambridge Philosophical Society 100, no. 1: 258–274. 39234845 10.1111/brv.13139 PMC11718601

[ece373159-bib-0014] Hoban, M. L. , J. Whitney , A. G. Collins , et al. 2022. “Skimming for Barcodes: Rapid Production of Mitochondrial Genome and Nuclear Ribosomal Repeat Reference Markers Through Shallow Shotgun Sequencing.” PeerJ 10: e13790. 35959477 10.7717/peerj.13790 PMC9359134

[ece373159-bib-0015] Jackman, J. M. , C. Benvenuto , I. Coscia , et al. 2021. “eDNA in a Bottleneck: Obstacles to Fish Metabarcoding Studies in Megadiverse Freshwater Systems.” Environmental DNA 3, no. 4: 837–849.

[ece373159-bib-0016] Jeunen, G. J. , E. Dowle , J. Edgecombe , U. von Ammon , N. J. Gemmell , and H. Cross . 2023. “Crabs‐A Software Program to Generate Curated Reference Databases for Metabarcoding Sequencing Data.” Molecular Ecology Resources 23, no. 3: 725–738. 36437603 10.1111/1755-0998.13741

[ece373159-bib-0017] Katoh, K. , and D. M. Standley . 2013. “MAFFT Multiple Sequence Alignment Software Version 7: Improvements in Performance and Usability.” Molecular Biology and Evolution 30, no. 4: 772–780. 23329690 10.1093/molbev/mst010 PMC3603318

[ece373159-bib-0018] Keck, F. , and F. Altermatt . 2023. “Management of DNA Reference Libraries for Barcoding and Metabarcoding Studies With the R Package Refdb.” Molecular Ecology Resources 23, no. 2: 511–518. 36239541 10.1111/1755-0998.13723

[ece373159-bib-0019] Keck, F. , J. Brantschen , and F. Altermatt . 2023. “A Combination of Machine‐Learning and eDNA Reveals the Genetic Signature of Environmental Change at the Landscape Levels.” Molecular Ecology 32, no. 17: 4791–4800. 37436405 10.1111/mec.17073

[ece373159-bib-0020] Keck, F. , M. Couton , and F. Altermatt . 2023. “Navigating the Seven Challenges of Taxonomic Reference Databases in Metabarcoding Analyses.” Molecular Ecology Resources 23, no. 4: 742–755. 36478393 10.1111/1755-0998.13746

[ece373159-bib-0021] Koster, J. , and S. Rahmann . 2012. “Snakemake—A Scalable Bioinformatics Workflow Engine.” Bioinformatics 28, no. 19: 2520–2522. 22908215 10.1093/bioinformatics/bts480

[ece373159-bib-0022] Kozlov, A. M. , J. Zhang , P. Yilmaz , F. O. Glöckner , and A. Stamatakis . 2016. “Phylogeny‐Aware Identification and Correction of Taxonomically Mislabeled Sequences.” Nucleic Acids Research 44, no. 11: 5022–5033. 27166378 10.1093/nar/gkw396 PMC4914121

[ece373159-bib-0032] Leary, N. A. , M. W. Wright , J. R. Brister , et al. 2016. “Reference Sequence (RefSeq) Database at NCBI: Current Status, Taxonomic Expansion, and Functional Annotation.” Nucleic Acids Research 44, no. D1: D733–D745. 26553804 10.1093/nar/gkv1189 PMC4702849

[ece373159-bib-0023] Meglecz, E. 2023. “COInr and mkCOInr: Building and Customizing a Nonredundant Barcoding Reference Database From BOLD and NCBI Using a Semi‐Automated Pipeline.” Molecular Ecology Resources 23, no. 4: 933–945. 36656075 10.1111/1755-0998.13756

[ece373159-bib-0024] Price, M. N. , P. S. Dehal , and A. P. Arkin . 2010. “FastTree 2—Approximately Maximum‐Likelihood Trees for Large Alignments.” PLoS One 5, no. 3: e9490. 20224823 10.1371/journal.pone.0009490 PMC2835736

[ece373159-bib-0025] Ratnasingham, S. , and P. D. Hebert . 2007. “The Barcode of Life Data System.” Molecular Ecology Notes 7, no. 3: 355–364. 18784790 10.1111/j.1471-8286.2007.01678.x PMC1890991

[ece373159-bib-0026] Redmond, A. K. , D. Casey , M. K. Gundappa , D. J. Macqueen , and A. McLysaght . 2023. “Independent Rediploidization Masks Shared Whole Genome Duplication in the Sturgeon‐Paddlefish Ancestor.” Nature Communications 14, no. 1: 2879. 10.1038/s41467-023-38714-z PMC10199039 37208359

[ece373159-bib-0027] Schenekar, T. , M. Schletterer , L. A. Lecaudey , and S. J. Weiss . 2020. “Reference Databases, Primer Choice, and Assay Sensitivity for Environmental Metabarcoding: Lessons Learnt From a Re‐Evaluation of an eDNA Fish Assessment in the Volga Headwaters.” River Research and Applications 36, no. 7: 1004–1013.

[ece373159-bib-0028] Steinegger, M. , and S. L. Salzberg . 2020. “Terminating Contamination: Large‐Scale Search Identifies More Than 2,000,000 Contaminated Entries in GenBank.” Genome Biology 21, no. 1: 115. 32398145 10.1186/s13059-020-02023-1 PMC7218494

[ece373159-bib-0029] Timm, L. E. , C. Tribuzio , R. P. Walter , et al. 2023. “Molecular Ecology of the Sleeper Shark Subgenus Somniosus (Somniosus) Reveals Genetic Homogeneity Within Species and Lack of Support for *S. antarcticus* .” Journal of Heredity 114, no. 2: 152–164. 36477342 10.1093/jhered/esac064

[ece373159-bib-0030] Toews, D. P. , and A. Brelsford . 2012. “The Biogeography of Mitochondrial and Nuclear Discordance in Animals.” Molecular Ecology 21, no. 16: 3907–3930. 22738314 10.1111/j.1365-294X.2012.05664.x

[ece373159-bib-0033] Weigand, H. , A. J. Beermann , F. Čiampor , et al. 2019. “DNA Barcode Reference Libraries for the Monitoring of Aquatic Biota in Europe: Gap‐Analysis and Recommendations for Future Work.” Science of the Total Environment 678: 499–524. 31077928 10.1016/j.scitotenv.2019.04.247

[ece373159-bib-0031] Xiong, F. , L. Shu , H. Zeng , X. Gan , S. He , and Z. Peng . 2022. “Methodology for Fish Biodiversity Monitoring With Environmental DNA Metabarcoding: The Primers, Databases and Bioinformatic Pipelines.” Water Biology and Security 1, no. 1: 100007.

